# Efficacy of Robot-Assisted Gait Therapy Compared to Conventional Therapy or Treadmill Training in Children with Cerebral Palsy: A Systematic Review with Meta-Analysis

**DOI:** 10.3390/s22249910

**Published:** 2022-12-16

**Authors:** Irene Cortés-Pérez, Noelia González-González, Ana Belén Peinado-Rubia, Francisco Antonio Nieto-Escamez, Esteban Obrero-Gaitán, Héctor García-López

**Affiliations:** 1 Department of Nursing, Physiotherapy and Medicine, University of Almería, Road Sacramento s/n, 04120 Almería, Spain; 2 Department of Health Sciences, University of Jaen, Campus las Lagunillas, 23071 Jaén, Spain; 3 “Torremar” Physiotherapy Center, Street Dr. Fleming 39, 29740 Torre del Mar, Spain; 4 AFIXA Fibromyalgia Association, 23008 Jaén, Spain; 5 Department of Psychology, University of Almería, Road Sacramento s/n, La Cañada, 04120 Almería, Spain; 6 Center for Neuropsychological Assessment and Neurorehabilitation (CERNEP), University of Almería, Road Sacramento s/n, La Cañada, 04120 Almería, Spain

**Keywords:** cerebral palsy, robot-assisted gait training, conventional therapy, treadmill training, gait, standing, movement abilities, functional independence, meta-analysis

## Abstract

Background: Motor, gait and balance disorders reduce functional capabilities for activities of daily living in children with cerebral palsy (CP). Robot-assisted gait therapy (RAGT) is being used to complement conventional therapy (CT) or treadmill therapy (TT) in CP rehabilitation. The aim of this systematic review is to assess the effect of RAGT on gait, balance and functional independence in CP children, in comparison to CT or TT. Methods: We have conducted a systematic review with meta-analysis. A search in PubMed Medline, Web of Science, Scopus, CINAHL, PEDro and SciELO has been conducted for articles published until October 2022. Controlled clinical trials (CCT), in which RAGT was compared to TT or CT and assessed gait speed, step and stride length, width step, walking distance, cadence, standing ability, walking, running and jumping ability, gross motor function and functional independence in children with CP, have been included. Methodological quality was assessed with the PEDro scale and the pooled effect was calculated with Cohen’s Standardized Mean Difference (SMD) and its 95% Confidence Interval (95% CI). Results: A total of 15 CCTs have been included, providing data from 413 participants, with an averaged methodological quality of 5.73 ± 1.1 points in PEDro. The main findings of this review are that RAGT shows better results than CT in the post-intervention assessment for gait speed (SMD 0.56; 95% CI 0.03 to 1.1), walking distance (SMD 2; 95% CI 0.36 to 3.65) and walking, running and jumping ability (SMD 0.63; 95% CI 0.12 to 1.14). Conclusions: This study shows that the effect of RAGT is superior to CT on gait speed, walking distance and walking, running and jumping ability in post-intervention, although no differences were found between RAGT and TT or CT for the remaining variables.

## 1. Introduction

Cerebral palsy (CP) is defined as a group of permanent motor and posture disorders that cause limitations in activity, resulting from non-progressive injuries that occurred in the developing fetal or infant brain [1,2,3]. Currently, CP is the most frequent cause of physical disability in childhood [4], reaching about 17 million cases in the world [5], which produces a large socio-economic burden. In 2013, its prevalence was 2.1 cases per 1000 live births [6]. However medical advancements and socioeconomic development have reduced the prevalence by about 25% to 1.6 per 1000 live births [7], although the prevalence varies according to the country’s development level [5,8]. The prevalence in high-income countries is about 1.6 cases per 1000 live births, while for middle-income countries it is doubled, reaching 3 cases per 1000 live births [9]. The etiology of CP is multifactorial, it is essential to know its specific origin for appropriate management, prognosis and rehabilitation [10]. Causes or risk factors involved in CP can be antenatal and perinatal (prematurity, lower birth weight, brain malformations, or brain hypoxia due to birth asphyxia) [11], or related to the mother (maternal obesity, unhealthy habits and lifestyle or infection during pregnancy) [12], prematurity and birth asphyxia being the most common causes [13]. Diagnosis is usually carried out between 12 and 24 months of life, through a clinical evaluation, assessing the presence of clinical risk factors and neuroimaging [14]. Latest advances in medical technology have allowed making a diagnosis at 6 months [8]. Generally, CP is classified according to its clinical symptoms as ataxic, hypotonic, dyskinectic and spastic, spastic CP being the most common diagnostic (85%–91%) [8]. In addition, signs and symptoms can appear unilaterally (monoparesis or hemiparesis), or bilaterally (diparesis, tripparesis and tetraparesis) [15].

CP implies the presence of different impairments, such as motor, sensory, cognitive, communicative, perceptual, behavioral, sleep or nutritional and digestive, among other deficits [16,17,18,19,20]. Motor disorders are the most common symptoms in these patients and are produced by damage in the motor brain cortex and the descending medullar pathways, particularly the corticospinal tract [21,22]. Muscle impairments due to spasticity or hypertonia, stiffness, weakness or hypotonia, reduced strength or muscle pain are some examples of these motor symptoms [23,24], which reduce joint range of motion, selective motor control and gross motor skills [25], impairing the performance of activities of daily living [26,27]. In addition, visual or vestibular disturbances produce gait [28] and balance disorders [29], which increase the risk of falls. On the one hand, gait is a motor function that makes possible mobility and exploration of the environment. It is closely linked to children’s functional capacity and quality of life. Thus, its alteration will affect different levels of child development [30]. Related to gait disorders, these patients present low gait speed, large individual support, short step length and step width and reduced cadence of gait, producing poor dynamic gait stability [31]. These gait impairments are compensated with an increased number of strides and wider stride width [32]. On the other hand, children with CP show important difficulties to maintain the balance while they are walking, running or in standing up position. Regarding their sitting abilities, these patients develop unsafe and inadequate posture due to the weakness of trunk muscles and poor back motor control [33].

The scientific literature published to date indicates that the most appropriate treatments to promote gross motor function in CP are based on functional therapy, which characterizes by the execution of movements proper of motor skills included in activities of daily living [34]. In the rehabilitation of gait and balance disorders in children with CP, classical passive conventional therapy and locomotor training using treadmills are the preferred therapies to restore gait and balance. However, in the last few years for the rehabilitation of gait, balance and functional upper extremity movements in children with CP, technological advances have introduced new devices such as virtual reality hardware and software, sophisticated balance platforms or kinematic sensors, such as Leap Motion Controller™ with valuable results [35,36,37,38,39]. Among these new technological approaches for rehabilitation in adulthood and childhood is the use of robotic devices [40,41], especially for gait training in patients with neurological deficits [42]. Robot-assisted gait therapy (RAGT) devices use an orthosis anchored to the body through an adjustable harness, which allows assisted walking on the treadmill [43]. These devices, as Lokomat^®^ as an example of one of the most used RAGT devices in these children, is provided by sensors in hips and knees joint that measure the human-machine interaction forces [44], adapting the movement and assisting the children in maintaining the gait trajectory [45]. This robotic technology allows longer training at variable speeds, while maintaining the same constant gait pattern [46]. Robotic devices allow and provide help to the leg movement during the gait cycle, and facilitate that brain receives sensory signals, which favors gait- and balance-related neuroplasticity [47]. RAGT is based on sensorimotor learning principles through intensive and repetitive simulation of different gait phases, along with visual and auditive stimulation. It also permits adjustments and modifications of the exercise, adapting the therapy to patient demands and needs [48], helping to reacquire a functional gait. Currently, it has acquired good acceptance in the clinical field regarding different neurological disorders such as spinal cord injury [49], stroke [50] or neurodegenerative processes such as Parkinson’s syndrome [51] or multiple sclerosis [52].

In the last 15 years, several studies have assessed the usability and effects of RAGT in children with CP, in comparison to CT or TT [53,54,55]. There is evidence from three previous published reviews showing that RAGT may be a safe and useful therapy to be used in the recovery of gait, balance and gross motor function disorders in these children. However, these reviews do not report substantial variation between RAGT and other therapies, such as CT or TT [56,57,58]. In 2021, Cumplido et al. conducted a systematic review that included 21 articles (only five were CP, mixed with spinal muscular atrophy, and not all of them were designed to compare two groups) published until 2020, in which the year of publication filter was included as well as the age of the participants [56]. Another study by Volpini et al. conducted a meta-analysis in 2022 including seven studies that analyzed the effect of RAGT on gross motor function and walking distance. This was the first meta-analysis suggesting that RAGT could be effective in the management of CP [57]. Finally, in 2022, Conner et al. published a meta-analysis including eight studies assessing the effectiveness of RAGT on gross motor function, walking distance and gait speed. However, their results did not find a significant difference across therapies (RAGT vs. others) [58]. However, these three reviews have some limitations that must be considered: (1) The number of studies published to date and the number of contributing participants was small, which reduces the robustness of the results; and (2) additional gait parameters such as step and stride length, width step or cadence have not been assessed to date. Taking into account that new studies have been published recently and the small number of studies included in the previous reviews, we have carried out a bibliographic search devoted to collecting all the scientific evidence available to date in order to assess the effect of RAGT (alone or combined with other therapies) on gait, balance, gross motor function and functional independence in CP children, compared to CT or TT. Additionally, we also want to assess the effect of RAGT according to different follow-up times (4–6 weeks and 2 and 3 months).

## 2. Materials and Methods

### 2.1. Study Design

This systematic review with meta-analysis has been carried out and reported following the recommendations of the *Preferred Reporting Items for Systematic Reviews and Meta-Analysis* (PRISMA) guidelines [59] and the *Cochrane Handbook for Systematic Reviews of Interventions* [60]. The methodology of this review was previously registered in the International Prospective Register of Systematic Reviews (PROSPERO), under the following number: CRD42022372589.

### 2.2. Source Data and Search Strategy

Two authors (I.C.-P. and H.G.-L.), independently, carried out a bibliographic search in PubMed Medline, Web of Science (WOS), Scopus, CINAHL Complete, PEDro (Physiotherapy Evidence Database) and SciELO up to October 2022. The retrieved full-text reference lists and gray literature were screened. A third author (E.O.-G.) was in charge of resolving discrepancies in this phase. For the search strategy, the PICOS tool proposed by the Cochrane Library [60] was used, identifying those studies susceptible to be included according to population (CP), intervention (RAGT), comparison (CT or TT), outcomes (gait, balance, gross motor function and functional independence) and study design (randomized and non-randomized controlled clinical trials (CCT)). The keywords “robotic”, “robotic gait assisted training” and “cerebral palsy” were used in the search strategy. Boolean operators “AND/OR” and specific tags were employed to combine these keywords for each database. No restrictions related to language, publication data and free-full text access were used. Table 1 shows the search strategies used for each database.

### 2.3. Study Screening: Inclusion and Exclusion Criteria

Two blinded authors (I.C.-P. and H.G.-L.), independently, reviewed the titles and abstracts of the records retrieved from databases. Doubts were resolved by a third author (E.O.-G.). All the studies selected by at least one of the investigators according to their title or abstract were then examined in detail for inclusion. Studies included in the review met all the following inclusion criteria: (1) CCT, pilot CCT or crossover CCT before to first crossover; (2) in which the effect of RAGT was analyzed; (3) compared to other interventions, such as CT or TT; (4) on the outcomes of interest (see Section 2.5); (5) in children with CP; and (6) providing quantitative data of the variables of interest after to finish the intervention. Exclusion criteria were (1) Non CCT; and (2) studies in which the sample included a range of neurological pathologies apart from CP and did not present their results disaggregated by pathology.

### 2.4. Data Extraction

This phase was carried out by two authors (N.G.-G. and A.B.P.-R.), who independently, extracted data from studies and compiled it in a standardized Excel sheet form. Disagreements were solved with the collaboration of a third author (E.O.-G.).

Data referred to: (1) the general characteristics of each study (authorship, study design, country and date of publication); (2) characteristics of the participants (study sample, number of groups, mean age, gender and type of CP); (3) characteristics of the intervention and control groups (type of therapy, type of RAGT device used, duration of the treatment in weeks, number of sessions per week and session duration); (4) post-intervention quantitative data of the variables of interest (mean and standard deviation); and (5) assessment time (post-intervention, follow-up 4–6 weeks and 2 and 3 months after to end the therapy). When a study did not provide standard deviations, they were calculated using standardized transformations through standard error, range, interquartile range and median [60,61].

### 2.5. Variables

The main variables analyzed in this systematic review were gait, gross motor function and functional independence. Related to gait, gait speed, step and stride length, width step, walking distance and cadence were assessed. Regarding gross motor function, we assessed the balance for standing position, walking, running and jumping and the total score of the gross motor function.

### 2.6. Methodological Quality and Quality of Evidence Assessment

The methodological quality of the included studies and the quality of evidence of their main findings were assessed by two authors (F.A.N.-E. and N.G.-G.), independently, and the doubts were consulted upon by a third author (H.G.-L.).

At first, the methodological quality of the included studies was assessed using the PEDro scale [62]. The PEDro scale is an 11-item checklist that can be scored as “yes” if the criterion is met and “no” otherwise. The total score can range from 0 (very low methodological quality and high risk of bias) to 10 (excellent methodological quality and very low risk of bias), while item 1 is not used to calculate the total score due to its relationship to external validity [62]. The methodological quality of a study is considered “excellent” if it reaches a score of 9 to 10 points; “good” for a score of 6 to 8 points; or “fair” for a score between 4 and 5 points; and “low quality” for a score lower than 3 [63].

On the other hand, we used the Grading of Recommendations Assessment, Development and Evaluation (GRADE) to assess the quality or level of evidence in the meta-analysis [64]. To assess the quality of evidence we took into account five items: risk of bias present in each study included, heterogeneity or inconsistency, indirect evidence, inaccuracy and risk of publication bias in each meta-analysis. All these, except the risk of bias in individual studies, were assessed using the GRADE checklist of Meader et al. (2014) [65]. The level of heterogeneity in each meta-analysis was used to assess the inconsistency (see Section 2.7). The precision was estimated taking into account the number of participants in each study (large > 300 participants, moderate 300–100 participants and low < 100 participants) and the number of studies included (large > 10 studies, moderate 10–5 studies and low < 5 studies). Indirect evidence was considered in those articles in which the results were indirectly measured [60]. Finally, the level of evidence can be: (1) high, when findings were robust; (2) moderate, if results might change after including new studies; (3) low, if the level of confidence in our original effect was very slight; and (4) very low, when some items from Meader’s checklist were not present.

### 2.7. Statistical Analysis

The meta-analysis was performed by two authors (E.O.-G. and I.C.-P.) using the software *Comprehensive Meta-Analysis version 3.0.* (Biostat, Englewood, NJ, USA, EE. UU.) [66]. To perform the meta-analysis for a variable, at least, two comparisons from one or more studies must be reported. The pooled effect was calculated using Cohen’s standardized mean difference (SMD) [67] and its 95% confidence interval (95% CI) according to the guidelines established by Cooper et al. [68] in a random effect of DerSimonian and Laird [69]. The pooled effect can be interpreted as a four-level effect: no effect (SMD 0), small (SMD 0.2), medium (SMD 0.5) and large (SMD > 0.8) [70]. The findings of each meta-analysis were displayed using the forest plots [71]. Red diamonds indicate the overall results of the meta-analysis. The center of the diamond corresponds to the overall effect value and its width represents the overall confidence interval. The difference between the intervention and control groups can be considered statistically significant if the diamond is clearly positioned to one side of the reference line, but if it overlaps or just touches the line, no conclusions can be drawn. The *p*-value for the Egger’s test (*p* < 0.1 indicating risk of publication bias) [72], the visualization of the funnel plot [73] (asymmetry indicates a possible risk of publication bias), and the trim-and-fill estimation [74] was used to estimate the risk of publication bias. When the trim-and-fill estimation reported a variation major than 10% with respect to the original SMD, the level of evidence was downgraded by one level [75]. The level of heterogeneity was calculated by using the Q-test and its *p*-value (*p* < 0.1 indicates the existence of heterogeneity) and the degree of Inconsistency (*I*^2^) established by Higgins [76]. So, the level of heterogeneity can be rated as low (*I*^2^ < 25%), moderate (*I*^2^ between 25–50%), or large (*I*^2^ > 50%) [77].

For each outcome, a meta-analysis was performed grouping the studies according to specific comparisons: RAGT vs. TT, RAGT vs. CT and RAGT plus CT vs. TT. The global effect including all the studies in the same group was not calculated due to the variability in the comparisons. Finally, subgroup analyses were performed for the follow-up assessments (4–6 weeks, 2 months and 3 months). Meta-regression was performed to assess differences in the pooled effect according to different study designs (randomized or non-randomized CCT).

## 3. Results

### 3.1. Search Results

**Figure 1:**
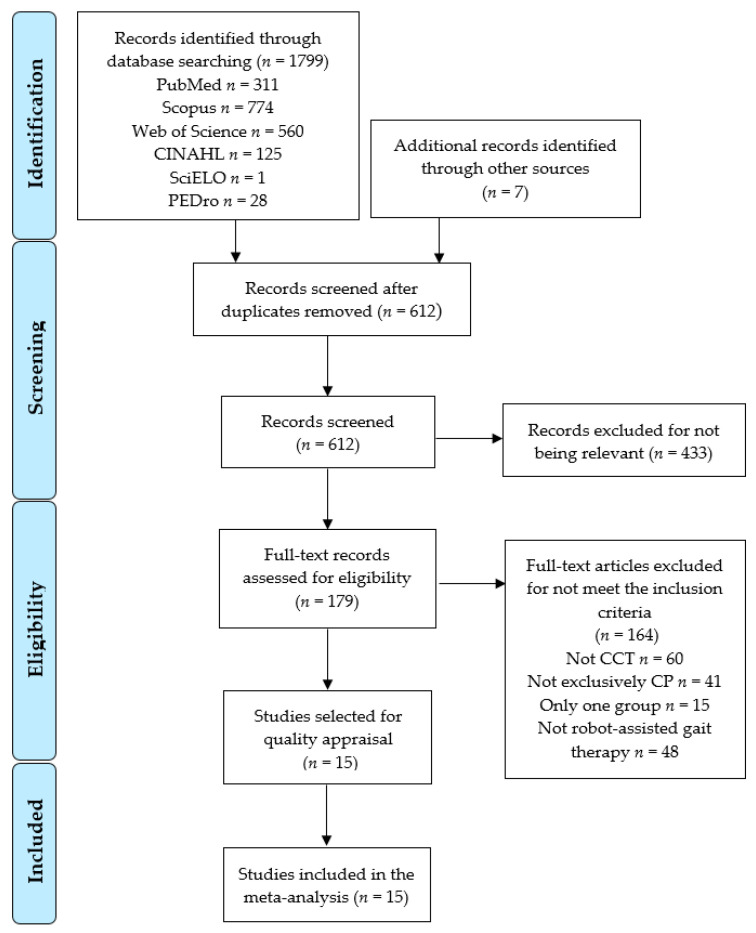
PRISMA Flow Diagram.

The initial search identified 1806 potential articles (PubMed Medline *n* = 311; SCOPUS *n* = 774; WOS *n* = 560; CINAHL Complete *n* = 125; SciELO *n* = 1; PEDro *n* = 28; and other sources *n* = 7). A total of 612 studies were screened by title and abstract and 433 were excluded for not being relevant. Later, 164 studies that did not meet the inclusion criteria were removed. Finally, 15 studies were included in the meta-analysis [78,79,80,81,82,83,84,85,86,87,88,89,90,91,92]. Figure 1 shows the PRISMA flow diagram corresponding to the study selection process.

### 3.2. Characteristics of the Included Studies

The included studies were carried out in Italy [80,89,91], France [81,82], Poland [85,88], Turkey [79,84], Slovakia [87], Switzerland [78], Australia, Saudi Arabia [92], Korea [86] and the United States [83] during the period between 2011 and 2021. Fourteen CCTs were randomized [78,79,80,81,82,83,85,86,87,88,89,90,91,92] and only one was non-randomized [84]. These studies provided data from 413 participants with a mean age of 10.33 ± 4.1 years old (185 girls and 228 boys). All subjects were diagnosed with unilateral (hemiplegic) and bilateral (diplegic, triplegic or tetraplegic) spastic CP. According to the Gross Motor Function Classification System (GMFCS), we collected data from participants in GMFCS I, II, III, IV and V, most of the cases being in GMFCS II, III and I (in this order). A total of 203 participants had been allocated to the experimental intervention groups receiving RAGT; while 210 participants were allocated to the control intervention group receiving CT or TT therapy. Table 2 shows, in detail, the characteristics of the studies included in this review. In the intervention group, the RAGT devices used were: Lokomat^®^ [78,79,81,82,85,87,89,91,92], Walkbot-K [86], EksoGT [88], RT600 [90], Gait Trainer GTI^®^ [80], 3DCaLT^®^ [83] and Innowalk Pro [84]. The duration of the intervention for each study is detailed in Table 3. Regarding the assessment time, all the studies reported data just at the end of the therapy (post-intervention), with 2 studies performing an additional follow-up between 4 and 6 weeks, other 2 studies at 2 months and 3 at 3 months. Finally, six of the studies included in this meta-analysis received external funding [78,86,87,88,90,92].

### 3.3. Methodological Quality of Included Studies

According to the PEDro scale, the mean score of the included studies was 5.73 ± 1.1, indicating a fair methodological quality. Thirteen studies included in this meta-analysis showed fair methodological quality [78,79,80,81,83,84,85,86,87,88,89,91,92], one study showed low methodological quality [82], and one study showed high methodological quality in this scale [90]. No study met items 5 and 6, which implies a large risk in performance and detection, respectively. Concealed allocation was not met in 11 studies, entailing a selection bias issue. Table 4 shows the score for each item on the PEDro scale.

### 3.4. Quantitative Synthesis

Ten outcomes were assessed in the meta-analysis gait speed, step length, width step, stride length, walking distance, cadence, standing up gross motor function, walking-running and jumping gross motor function, total gross motor function and functional independence. Table 5 shows the main findings of the meta-analysis.

#### 3.4.1. Gait Speed

**Figure 2:**
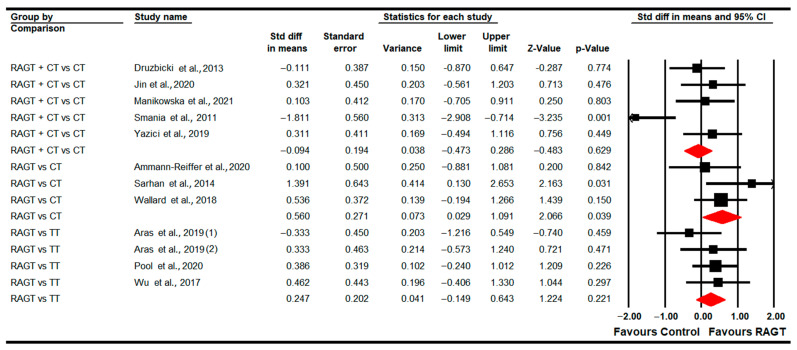
Forest Plot of Gait Speed (Post-Intervention Assessment) [78,79,80,82,83,84,85,86,88,90,92].

Eleven studies [78,79,80,82,83,84,85,86,88,90,92] provided data to assess the effect of RAGT on gait speed in the post-intervention assessment (just at the end of the intervention). Three studies with four independent comparisons provided data to compare RAGT vs. TT [79,83,90]; three studies with three independent comparisons compared RAGT vs. CT [78,82,92]; and five studies with five independent comparisons compared RAGT plus CT vs. CT [80,84,85,86,88]. Our findings showed statistically significant differences favoring RAGT (SMD 0.56; 95% CI 0.03 to 1.1; *p* = 0.04) in comparison to CT (Table 5, Figure 2). Not statistically significant differences were found between RAGT and TT (SMD 0.25; 95% CI −0.15 to 0.64; *p* = 0.22) and between RAGT plus CT and CT (SMD −0.1; 95% CI −0.47 to 0.29; *p* = 0.63). Heterogeneity and risk of publication were not found when RAGT and CT were compared (details in Table 5). Sensitivity analysis did not show substantial variations. No differences were found in meta-regression in the comparison of RAGT plus CT vs. CT.

Subgroups analysis revealed that 4 or 6 weeks after the end of the intervention RAGT plus CT is more effective (SMD −0.77; 95% CI −0.19 to 1.55; *p =* 0.067) than CT. Two months later, no differences were found between RAGT and TT (SMD 0.26; 95% CI −0.25 to 0.77; *p =* 0.32).

#### 3.4.2. Step Length

**Figure 3:**
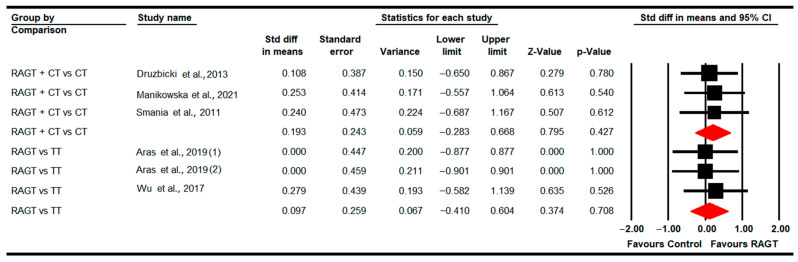
Forest Plot of Step Length (Post-Intervention Assessment) [79,80,83,85,88].

Five studies [79,80,83,85,88] assessed the effect of RAGT on step length at post-intervention time. Three studies with three independent comparisons compared RAGT plus CT vs. CT [80,85,88] and two studies with three independent comparisons, compared RAGT vs. TT [79,83]. No statistically significant differences were found between RAGT and TT (SMD 0.1; 95% CI −0.41 to 0.6; *p =* 0.71), and between RAGT plus CT and CT (SMD 0.2; 95% CI −0.28 to 0.67; *p =* 0.43) (Table 5, Figure 3). No heterogeneity nor risk of publication bias was found for any comparison (details in Table 5).

At 4–6 weeks, no differences were found between RAGT plus CT vs. CT (SMD 0.34; 95% CI −0.27 to 0.95; *p =* 0.28). However, at 2 months follow-up, subgroup analysis reported statistically significant differences in favor of RAGT vs. TT (SMD 0.88; 95% CI 0.32 to 1.43; *p =* 0.002).

#### 3.4.3. Step Width

**Figure 4:**
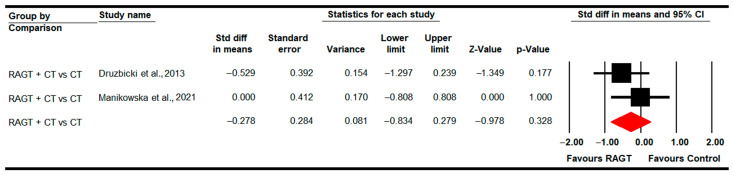
Forest Plot of Step width (Post-Intervention Assessment) [85,88].

Two studies [85,88] with two independent comparisons provided data to assess the effect of RAGT plus CT vs. CT in step width in the post-intervention assessment. No statistically significant differences were found between both therapies (SMD −0.28; 95% CI −0.83 to 0.28; *p =* 0.33) (Table 5, Figure 4). Heterogeneity was not present and the risk of publication bias could not be calculated (details in Table 5).

#### 3.4.4. Stride Length

**Figure 5:**
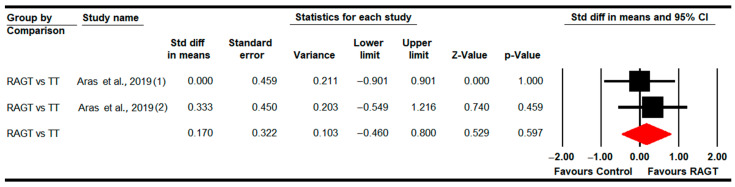
Forest Plot of Step width (Post-Intervention Assessment) [79].

One study [79] with two independent comparisons assessed the effect of RAGT, in comparison to TT, on stride length in the post-intervention assessment. Our findings did not show statistically significant differences between therapies (SMD 0.17; 95% CI −0.46 to 0.8; *p =* 0.6) (Table 5, Figure 5). Heterogeneity was not present and the risk of publication bias could not be calculated (details in Table 5).

#### 3.4.5. Walking Distance

**Figure 6:**
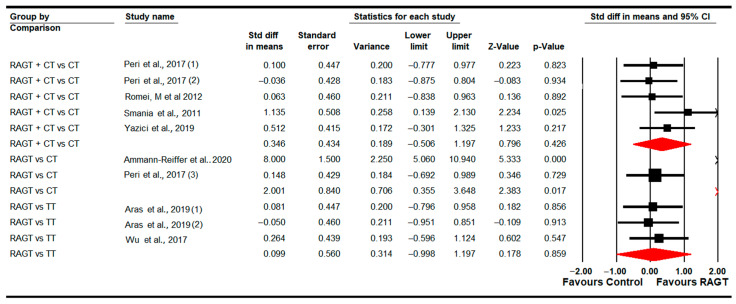
Forest Plot of Walking Distance (Post-Intervention Assessment) [78,79,80,83,84,89,91].

Seven studies [78,79,80,83,84,89,91] provided data to assess the effect of RAGT in the post-intervention assessment. Two studies with two independent comparisons provided data from the RAGT vs. CT comparison [78,89]; other two with three independent comparisons for RAGT vs. TT [79,83], and four studies with five independent comparisons for RAGT plus CT vs. CT [80,84,89,91]. Our findings showed statistically significant differences (SMD 2; 95% CI 0.36 to 3.65; *p =* 0.017) favoring RAGT in comparison to CT (Table 5, Figure 6). However, no statistically significant differences were found between RAGT and TT (SMD 0.1; 95% CI −1 to 1.2; *p =* 0.86), and RAGT plus CT vs. CT (SMD 0.35; 95% CI −0.51 to 1.2; *p =* 0.43). Moderate heterogeneity was present in the RAGT vs. CT meta-analysis (details in Table 5). Meta-regression did not report differences.

Subgroup analyses revealed no statistically significant differences between RAGT vs. TT (SMD 0.02; 95% CI −0.49 to 0.53; *p =* 0.941) at 2 months follow-up. At 3 months follow-up, no statistically significant differences were found between RAGT plus CT and CT (SMD 0.07; 95% CI −0.36 to 0.5; *p =* 0.75).

#### 3.4.6. Cadence

**Figure 7:**
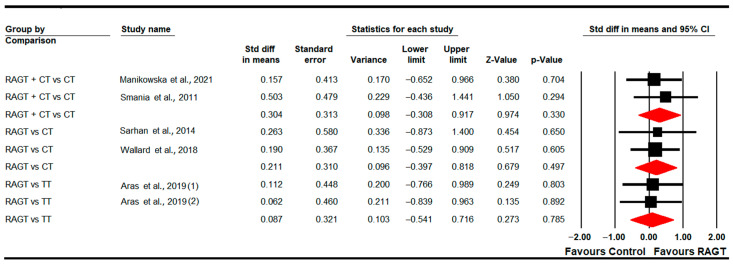
Forest Plot of Cadence (Post-Intervention Assessment) [79,80,82,88,92].

Five studies [79,80,82,88,92] provided data to assess the effect of RAGT on cadence post-intervention. Two studies with two independent comparisons provided data from RAGT vs. CT [82,92]; only one study [79] with two independent comparisons for RAGT vs. TT; and finally other two studies with two independent comparisons for RAGT plus CT vs. CT [80,88]. No statistically significant differences were found for RAGT vs. CT (SMD 0.21; 95% CI −0.4 to 0.82; *p =* 0.5), RAGT vs. TT (SMD 0.09; 95% CI −0.54 to 0.72; *p =* 0.79) and RAGT plus CT vs. CT (SMD 0.3; 95% CI −0.31 to 0.92; *p =* 0.33) (Table 5, Figure 7). Heterogeneity was not present (details in Table 5).

At 4–6 weeks follow-up, no statistically significant differences were found between RAGT plus CT and CT (SMD −0.06; 95% CI −0.67 to 0.55; *p =* 0.85). At 2 months follow-up, no differences were reported between RAGT and TT (SMD 0.11; 95% CI −0.52 to 0.74; *p =* 0.73).

#### 3.4.7. Standing Ability (GMFM-D Dimension)

**Figure 8:**
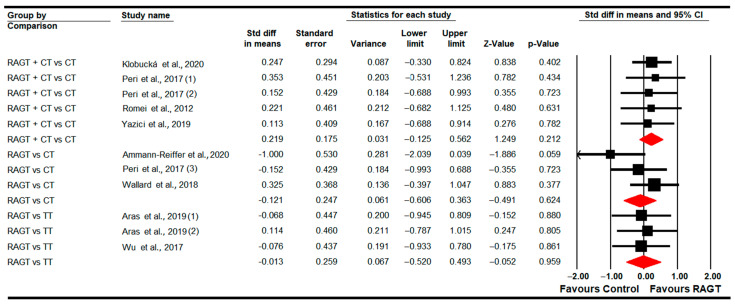
Forest Plot of Standing Ability (Post-Intervention Assessment) [78,79,81,83,84,87,89,91].

Eight studies [78,79,81,83,84,87,89,91] reported data to assess the effect of RAGT on standing ability. Three studies with three independent comparisons provided data on RAGT vs. CT [78,81,89]; two studies with other three independent comparisons regarding RAGT vs. TT [79,83]; and finally four studies with five independent comparisons for RAGT plus CT vs. CT [84,87,89,91]. No statistically significant differences were found between RAGT and CT (SMD −0.12; 95% CI −0.61 to 0.36; *p =* 0.62) and TT (SMD −0.01; 95% CI −0.52 to 0.5; *p =* 0.96), respectively; and between RAGT plus CT and CT (SMD 0.22; 95% CI −0.13 to 0.56; *p =* 0.21) (Table 5, Figure 8). Heterogeneity and risk of publication bias were only present in the RAGT vs. CT meta-analysis (details in Table 5). No differences were reported in meta-regression.

No statistically significant differences between RAGT and TT were found (SMD −0.01; 95% CI −0.52 to 0.49; *p =* 0.94) at 2 months follow-up; and between RAGT plus CT and CT (SMD −0.15; 95% CI −0.28 to 0.57; *p =* 0.5) at 3 months follow-up.

#### 3.4.8. Walking, Running and Jumping Ability (GMFM-E Dimension)

**Figure 9:**
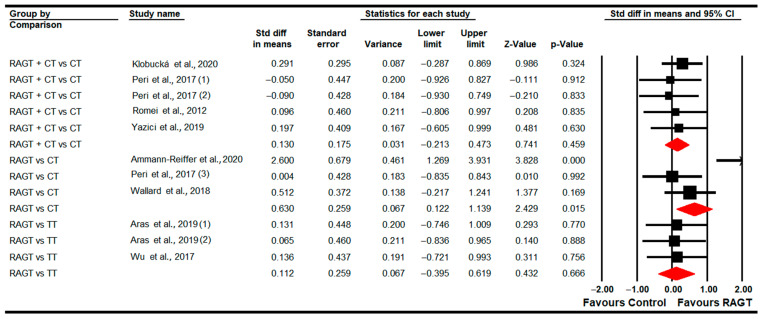
Forest plot of walking, running and jumping ability (Post-Intervention Assessment) [78,79,81,83,84,87,89,91].

Eight studies [78,79,81,83,84,87,89,91] reported data to analyze the effect of RAGT on walking, running and jumping abilities (assessed with the GMFM-E Dimension) at post-intervention. Three studies with three independent comparisons provided data for RAGT vs. CT [78,81,89]; two studies with three independent comparisons for RAGT vs. TT [79,83]; and finally, four studies with five independent comparisons for RAGT plus CT vs. CT [84,87,89,91]. Our findings showed a greater improvement following RAGT (SMD 0.63; 95% CI 0.12 to 1.14; *p =* 0.015) than CT (Table 5, Figure 9). No statistically significant differences were found between RAGT and TT (SMD 0.11; 95% CI −0.4 to 0.62; *p =* 0.67) and between RAGT plus CT and CT (SMD 0.13; 95% CI −0.21 to 0.47; *p =* 0.46). Heterogeneity was only present in the comparison between RAGT and TT, and the risk of publication bias was present in RAGT plus CT vs. CT (details in Table 5). Meta-regression did not show differences in pooled effect.

Subgroup analysis reported that at 2 months follow-up, no statistically significant differences were present between RAGT and TT (SMD 0.16; 95% CI −0.35 to 0.7; *p =* 0.53), nor at 3 months follow-up between RAGT plus CT vs. CT (SMD 0.02; 95% CI −0.41 to 0.44; *p =* 0.92).

#### 3.4.9. Gross Motor Function (Total Score)

**Figure 10:**
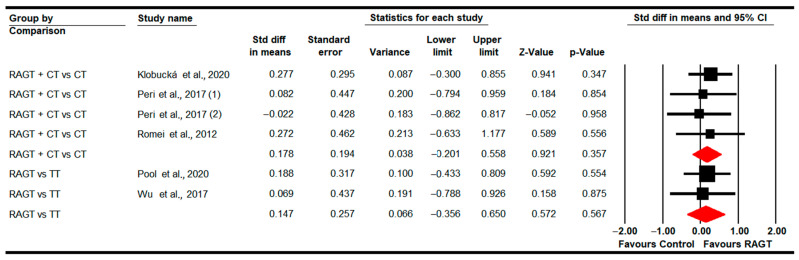
Forest Plot of Total Gross Motor Function (Post-Intervention Assessment) [83,87,89,90,91].

Five studies [83,87,89,90,91] provided data to assess the effect of RAGT on total gross motor function in the post-intervention assessment. Two studies with two independent comparisons reported data for RAGT vs. TT [83,90], and three studies with four independent comparisons for RAGT plus CT vs. CT [87,89,91]. The meta-analysis did not show statistically significant differences between RAGT and TT (SMD 0.15; 95% CI −0.36 to 0.65; *p =* 0.57) and between RAGT plus CT and CT (SMD 0.18; 95% CI −0.25 to 0.56; *p =* 0.36) (Table 5, Figure 10). Heterogeneity was not present and there is a potential risk of publication bias in the RAGT plus CT vs. CT comparison (details in Table 5).

At 3 months follow-up, no statistically significant differences were found between RAGT plus CT and CT (SMD −0.14; 95% CI −0.64 to 0.37; *p =* 0.6).

#### 3.4.10. Functional Independence

**Figure 11:**
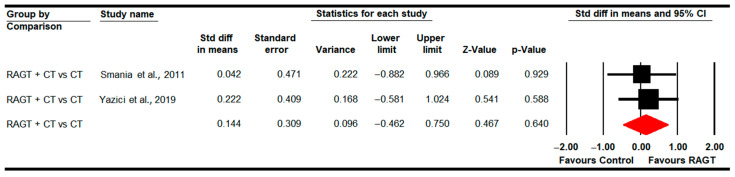
Forest Plot of Functional Independence (Post-Intervention Assessment) [80,84].

Two studies [80,84] with two independent comparisons assessed the effect of RAGT plus CT vs. CT on functional independence in the post-intervention. No statistically significant differences were found between therapies (SMD 0.14; 95% CI −0.46 to 0.75M *p =* 0.64) (Table 5, Figure 11). Heterogeneity was not present and the risk of publication bias could not be calculated (details in Table 5). No differences were found in meta-regression.

## 4. Discussion

Motor disabilities experienced by children with CP, such as muscle tone disorders, stiffness, decreased joint range of motion and poor trunk motor control, among others, cause a reduction of their functional mobility, quality of life and personal autonomy or independence [93]. Three functions highly affected are gait ability, the capability to maintain posture in seating and standing positions and gross motor skills [94,95,96]. Once children receive a diagnosis of CP it is necessary to carry out an early care treatment adapted to the characteristics of their pathology. This treatment will be provided by clinicians, parents or professional caregivers during their lifetime [97]. Physiotherapy-based conventional approaches and locomotor training using treadmills are the most usual methods administered for the recovery of gait, balance, gross motor functional and independence in these patients. Although these approaches have resulted in being effective in these patients, they are passive techniques and sometimes patients report a lack of motivation and monotony. In order to favor the active participation of the patient in the therapy, some technological solutions have been used in the last years. One of these advances relies on the use of robotic devices that assist patients in gait recovery or the development of activities of daily living. Numerous original studies have assessed the effect of RAGT in these patients, reporting a positive effect in gait and balance recovery. However, there is no consensus on whether it is a better therapeutic option than CT or TT. Thus, the aim of this systematic review with meta-analysis was to retrieve all the scientific evidence available to date to check if RAGT (used as isolated therapy or combined with CT or TT) is more effective than CT or TT for improving gait ability, gross motor function and functional independence in children with CP. In addition, we wanted to determine if the effect of RAGT was maintained over time. After performing the meta-analysis, our findings showed that RAGT can be better than CT for improving gait speed, walking distance and walking-running and jumping ability in CP children.

To date, four recent systematic reviews have assessed the effect of RAGT on gait, gross motor and functional disability in children with CP [56,57,58,98]. Carvalho and colleagues, in 2017, performed a meta-analysis with 10 studies (including observational and experimental designs) to assess the effect of RAGT vs. other therapies, without reporting statistically significant differences for gait speed, walking distance and gross motor function [98]. Cumplido and colleagues compiled 21 studies with different designs, such as case reports, case series, quasi-experimental studies and RCT, which assessed the effect of RAGT in children with CP and spinal muscular atrophy [56]. Regarding CP, RAGT seemed to be safe and the qualitative analysis reported positive results. Later, Volpini and colleagues assessed the effect of RAGT on gait speed, walking distance and the D and E dimensions of gross motor function collecting data from 7 studies and 77 individuals [57]. However, they did not perform specific comparisons, as has been done in the current review. They assessed the improvement between pre- and post-RAGT, and in a long-term follow-up, showing that RAGT can be useful to improve waking distance in the short-term, but they did not inform if RAGT is more effective than other therapies. Finally, Conner and colleagues compiled 8 RCTs providing data from 188 individuals with CP to assess if RAGT is more effective than other therapies. These authors did not discriminate the type of control therapy [58]. Their findings showed that RAGT was not better than control therapies in improving walking distance, gait speed and the D and E dimensions of gross motor function. It is not easy to compare our results with the previous reviews as our analysis has considered specific control comparisons, the meta-analysis by Carvalho and Corner is the most similar to our study. In addition, previous reviews have only considered variables such as walking distance, gait speed or the D and E gross motor dimensions, neglecting that gait is a complex ability and it is important to assess other parameters such as cadence, step and stride length, width step or functional independence, variables that we have assessed here. Therefore, our systematic review differentiates itself from previous reviews and completes a gap of knowledge about the effect of RAGT in children with CP, compared to the classical approaches (CT or TT). An additional strength of our review is that the current meta-analysis has included the larger number of CCT published to date, a total of 15 studies including data from 403 participants, which shows the interest of the authors to include all the existing studies and reduce the possibility of risk of publication bias in the review.

Our meta-analysis has also assessed a larger number of gait-related parameters, such as step and stride length, width step or cadence, apart from classical outcomes like gait speed and walking distance. Compared to classical CT, our findings show a large effect in favor of RAGT in improving gait speed, walking distance and walking-running and jumping ability (E dimension of gross motor function) in patients with CP just at the end of the therapy (post-intervention). These findings disagree with Carvalho (2017) and Conner (2022), who did not obtain statistically significant differences between RAGT and other therapies [58,98]. However, in our study, we did not find differences between RAGT and CT in improving step and stride length, width step, cadence or standing ability (D dimension of gross motor function). The improvements observed in gait speed, walking distance and the E dimension of gross motor function, in comparison to CT, are clinically relevant regarding the personal autonomy of CP children. The RAGT physical exercise approach improves aerobic capacity and increases gait speed and walking distance [99], which allows for performing more activities of daily living requiring movement without getting tired and without needing so much assistance from relatives or caregivers. This meta-analysis provides relevant evidence regarding RAGT as the better therapeutic option, compared to CT, for the recovery of the gait speed and dynamic balance associated with locomotion.

Regarding the comparison between RAGT and TT, our findings showed differences between these therapies in favor of RAGT for step length at a two-month follow-up. No differences were found at post-intervention time for any variable. Moreover, these findings cannot be compared with previous reviews as this is the first one that has specifically assessed the efficacy of RAGT vs. TT. Therefore, we suggest that the main effect of RAGT with respect to classical TT appears at mid-term, although it is not possible to confirm if this effect is maintained for more time because no studies have compared the effect of RAGT vs. TT after two months. Nevertheless, these findings must be considered with caution due to the small number of studies included in the review, which reduces the accuracy and quality of the evidence. The current findings can be explained by the similarity between RAGT and TT therapies. Both require the active participation of the patients and, in both cases, they receive external support, either from the robot or from a suspended load. The only difference between them is that RAGT helps to perform the movement, whereas, in TT, it is the subject who starts and stops the movement, increasing fatigue and reducing efficacy.

One interesting finding of this review is that it suggests that the use of RAGT combined with CT is not superior to CT alone in improving gait, gross motor function and functional independence. Sometimes, the combination of two or more therapies in patients with large disabilities can increase muscle fatigue, making it difficult to carry out the therapy, creating a feeling of frustration in these patients, a consequence of not achieving any improvement in the trained skills. However, it is necessary to point out that these results present low levels of evidence and precision, especially regarding the functional independence variable, due to the small number of comparisons per meta-analysis and the risk of publication bias that could underestimate the results against RAGT.

A possible explanation for the lack or reduced difference among the analyzed therapies is the heterogeneity of treatment protocols, as there is uniformity in the number of sessions and time per session. This made difficult the comparison among studies, and subgroup analysis according to the treatment dose was considered. However, in some cases, the number of comparisons by meta-analysis was only one, which would not provide further evidence than the particular study, with very low-quality evidence. Finally, most of the published literature included Lokomat^®^ as the gait assistant robot device, as do most of the studies included in the present review. Therefore, this device can be considered the most effective robot assistance device to be used in the gait rehabilitation of CP children. Lokomat^®^ is a suitable and safe robot device used in pediatric patients, made by a treadmill, a patient suspension system and lower extremity orthoses electronically controlled by a specific software that synchronizes the treadmill gait, the body weight support and the movement of lower limb orthoses according to the characteristics of each patient, which permits the personalization of the therapy [100].

The improvements produced by RAGT can be due to different reasons. Firstly, RAGT facilitates patients’ alternate step movements, which would reproduce patterns of physiological muscle activation [101] and increase motor learning as a result of a task-specific repetitive approach [102]. A recent study, published in November 2022 by Perpetuini and colleagues, showed that RAGT produces modifications in the motor and pre-frontal brain cortex, improving motor control and attention during RAGT in children with CP [103], showing that neuroplasticity is essential in the recovery of these patients. For motor control improvements, RAGT produced bilateral changes in cortical areas BA 1, 6, 9, 11 and 46, which are involved in motor coordination and complex movements (BA 6 and 9), proprioceptive control (BA 1), spatial memory (BA 9) and in attention, self-control and working memory (BA 9 and 46). It could result in higher scores in gross motor function measurement and its sub-dimensions related to gait and balance. Secondly, RAGT requires active and total participation of children in the therapy and can be used in combination with other activities of daily living that need standing balance training. Moreover, active participation involves attention and engagement in the therapy, which would involve an increase of activity in the pre-frontal cortex [103]. RAGT seems to improve cardiopulmonary function, allowing patients to perform activities for more time without experiencing fatigue or tiredness [104]. Finally, one of the future objectives is to reduce the energy expenditure when these patients walk with disabilities or train their gait using RAGT. In this line, recent studies estimate the gait expenditure during RAGT by collecting data from different reliable, ergonomic and validity body sensors, such as heart rate and inertial wearable sensors [105]. Combining reliable sensors with new RAGT devices could increase the effectiveness of this therapy in these children.

Lastly, although the results reported in this systematic review are of interest in terms of clinical practice, some limitations must be commented on. First, it is necessary to highlight the small number of studies included in each meta-analysis, which reduces the generalizability of our findings. Second, the impossibility of blinding participants and assessors could have distorted the true effect of the therapy and reduced the accuracy of our findings. Third, the risk of publication bias in some meta-analyses, above 10% in Trim-and-fill estimation, could have underestimated the original effect. Another limitation is that the follow-up assessment was performed in just a few studies, and it is necessary to assess the effect of RAGT over time. Future research must be performed to assess the effect of RAGT on gait, balance, gross motor function and functional independence in these children, with the aim of gaining findings more robust and generalizable.

## 5. Conclusions

This is the first meta-analysis that analyzes and provides evidence about the efficacy of RAGT in comparison to CT or TT. Our results did not show a clear superiority for RAGT (alone or combined with CT) with regard to CT or TT. This meta-analysis has shown that RAGT is more effective than CT in improving gait speed, walking distance and walking-running and jumping abilities (E dimension of gross motor function), just at the end of the therapy (post-intervention). RAGT only seems to be superior to TT in improving step length at the 2-month follow-up. RAGT was not superior to CT or TT at post-intervention regarding step and stride length, width step, cadence, standing ability, global gross motor function and functional independence. However, these results must be considered with caution independently of their statistical significance, due to the small number of studies and comparisons included in the meta-analysis, with the level of evidence being low. In future research, it is necessary to carry out new RCTs that compare the efficacy of these therapies. This will increase the level of evidence of these results, reducing, as much as possible, performance, detection and selection biases observed in the studies included here.

## Figures and Tables

**Table 1:** Search strategies in databases.

Database	Search Strategy
PubMed Medline	(cerebral palsy [mh] OR cerebral palsy [tiab]) AND (robot [tiab] OR robotic [tiab] OR exoskeleton [tiab] OR robotic gait assisted training [tiab])
SCOPUS	TITLE-ABS-KEY (“cerebral palsy”) AND TITLE-ABS-KEY (“robotic” OR “exoskeleton” OR “robot gait assisted training”)
Web of Science	TOPIC: (*cerebral palsy*) AND TOPIC: (*robotic* OR *exoskeleton* OR *robot gait assisted training*)
CINAHL Complete	AB (cerebral palsy) AND AB (robotic OR exoskeleton OR robot gait assisted training)
PEDro	cerebral palsy AND robotic cerebral palsy AND robot
SciELO	cerebral palsy AND robotic

**Table 2:** Characteristics of the studies included in the systematic review and meta-analysis.

Study	Funding	N	F/M	CP Type	TC	GMFCS	Groups	Age (Years)	Evaluation	Outcomes	Test
Ammann–Reiffer, C et al., 2020 (Switzerland) [78]	Yes	16	3/13	Spastic	Bilateral	II *n* = 9 III *n* = 5 IV *n* = 2	CG *n* = 8 EG *n* = 8	11.3 ± 2.3	T_1_ post-intervention	Gait speed Gross motor function Walking distance	10MWTS GMFM-88 D, E 6MWT
Aras, B et al., 2019 (Turkey) [79]	No	29	11/18	Spastic	Hemiplegic *n* = 9 Diplegic *n* = 20	II *n* = 24 III *n* = 5	CG_1_ *n* = 10 CG_2_ *n* = 9 EG *n* = 10	9.3 ± 2.3	T_1_ post-intervention T_2_ Follow-up (2 months)	Gait speed Gross motor function Stride length Walking distance Cadence	3D gait analysis GMFM-66 D, E m 6MWT Step/min
Druzbicki, M et al., 2013 (Poland) [85]	No	35	19/16	Spastic	Diplegic	II *n* = 23 III *n* = 12	CG *n* = 9 EG *n* = 26	10.6 ± 2.3	T_1_ post-intervention	Step length Gait speed Step width	M m/s m
Jin, LH et al., 2020 (Korea) [86]	Yes	20	7/13	Spastic *n* = 17 Dyskinetic *n* = 1 Mixed *n* = 2	Hemiplegic *n* = 1 Diplegic *n* = 19	II *n* = 5 III *n* = 9 IV *n* = 6	CG *n* = 10 EG *n* = 10	6.8 ± 2.2	T_1_ post-intervention	Gait speed	m/s
Klobucká, S et al., 2020 (Slovakia) [87]	Yes	47	20/27	Spastic	Diplegic	I *n* = 1 II *n* = 7 III *n* = 21 IV *n* = 18	GC *n* = 26 GE *n* = 21	21.2 ± 5.3	T_1_ post-intervention	Gross motor function	GMFM-88, D, E and total
Manikowska, F et al., 2021 (Poland) [88]	Yes	26	10/16	Spastic	Bilateral	I-II *n* = 17 III-IV *n* = 9	CG *n* = 17 EG *n* = 9	14.8 ± 1.9	T_1_ post-intervention T_2_ post-intervention (6 weeks)	Gait speed Cadence Step width Step length	m/s Step/min m m
Peri, E et al., 2017 (Italy) [89]	No	44	22/22	Spastic	Bilateral	I *n* = 14 II *n* = 16 III *n* = 14 III *n* = 16	CG_1_ *n* = 10 EG_1_ *n* = 12 EG_2_ *n* = 10 EG_3_ *n* = 12	8.7 ± 1.7	T_1_ post-intervention T_2_ post-intervention (3 months)	Gross motor function	GMFM-88 D, E and total
										Walking distance	6MWT
Pool, D et al., 2021 (Australia) [90]	Yes	40	18/22	NR	NR	III *n* = 16IV *n* = 10 V *n* = 14	CG *n* = 20 EG *n* = 20	5–12 (range)	T_1_ post-intervention	Gross motor function Gait speed Functional Independence	GMFM-88 total 10MWTS WeeFIM
Romei, M et al., 2012 (Italy) [91]	No	19	11/8	Spastic	Bilateral	I *n* = 6 II *n* = 11 III *n* = 3	CG *n* = 10 EG *n* = 9	8.1 ± 1.7	T_1_ post-intervention T_2_ post-intervention (3 months)	Gross motor function	GMFM-88 D, E and total
										Walking distance	6MWT
Sarhan, RSM et al., 2014 (Saudi Arabia) [92]	Yes	12	5/7	Spastic	Diplegic	III-IV	CG *n* = 6 EG *n* = 6	4.2 ± 0.7	T_1_ post-intervention	Cadence Gait speed Stride length	step/min m/s m
Smania, N et al., 2011 (Italy) [80]	No	18	8/10	Spastic	Diplegic *n* = 11 Tetraplegic *n* = 7	I *n* = 6 II *n* = 2 III *n* = 3 IV *n* = 7	CG *n* = 9 EG *n* = 9	12.5 ± 2.9	T_1_ post-intervention T_2_ post-intervention (1 month)	Gait speed Walking distance Cadence Step length Functional Independence	m/s 6MWT step/min m WeeFIM
Wallard, L et al., 2017 (France) [81]	No	30	15/15	Spastic	Diplegic	II	CG *n* = 16 EG *n* = 14	8.9 ± 1.4	T_1_ post-intervention	Gross Motor Function	GMFM-88 D, E
Wallard, L et al., 2018 (France) [82]	No	30	15/15	Spastic	Diplegic	II	CG *n* = 16 EG *n* = 14	8.9 ± 1.4	T_1_ post-intervention	Gait speed Cadence Step length Step width	m/s step/min m m
Wu, M et al., 2017 (United States) [83]	No	23	9/14	Spastic	Diplegic *n* = 11 Triplegic *n* = 1 Tetraplegic *n* = 7	I *n* = 3 II *n* = 9 III *n* = 8 IV *n* = 3	CG *n* = 12 EG *n* = 11	10.9 ± 3.2	T_1_ post-intervention T_2_ post-intervention (2 months)	Gait speed Gross Motor Function	m/s GMFM 88 D, E, total
										Step length Walking distance	M 6MWT
Yazici, M et al., 2019 (Turkey) [84]	No	24	12/12	Spastic	Hemiplegic	I-II	CG *n* = 12 EG *n* = 12	8.5 ± 8.5	T_1_ post-intervention T_2_ post-intervention (3 months)	Gait speed Gross Motor FunctionWalking distance Functional Independence	10MWTS GMFM 88 D, E6MWT FAQ-WL

Abbreviations: CG, Control Group; CP, Cerebral Palsy; D, Dimension D (Standing); E, Dimension E (Walking, running and jumping); EG, Experimental Group; F, Females; GMFCS, Gross Motor Function Classification System; GMFM, Gross Motor Function Measure; K, Number of Comparisons; m, Meters; M, Males; min, Minutes; N, Number of Participants; RTC, Randomized Controlled Trial; TC, Topographical Classification; 10MWTS, Ten-meter walking test; 6MWT, Six minutes walking test; WeeFIM, Wee Functional Independence Measure; FAQ-WL, Functional Assessment Questionnaire Walking Scale; NR, Not reported.

**Table 3:** Characteristics of the studies included in the systematic review and meta-analysis.

Study	Intervention	Type Robot	Session Time (min)	Number of Sessions	Frequency (ss/wk)	Duration of Treatment (wk)	Qualitative Findings
Ammann-Reiffer, C et al., 2020 [78]	CG UC (CT) EG RAGT	Lokomat^®^	45	35 25	2/3/2 3/2	5/5/5 5/5	No significant differences were found after the RAGT period in dimensions E (*p* = 0.91), D (*p* = 0.46) and gait speed.
Aras, B et al., 2019 [79]	CG_1_ PBWSTE (TT) CG_2_ ATE (TT) EG RAGT	Lokomat^®^	45	20	5	4	No statistically significant difference among the groups according to the GFMF-D, GMFM-E and 6MinWT (*p* > 0.05).
Druzbicki, M et al., 2013 [85]	CG IE (CT) EG RAGT + IE	Lokomat^®^	45	20	5	4	Improvement of both groups in gait speed with no significant difference between groups (*p* = 0.5909). Decrease in range of motion with no significant difference between groups (*p* = 0.8676).
Jin, LH et al., 2020 [86]	CG CT EG RAGT + CT	Walkbot-K system^®^	30	36 54	3/3 3/3/3	12 18	No significant differences were found after the RAGT period in gait speed (*p* = 0.223).
Klobucká, S et al., 2020 [87]	CG CT EG RAGT	Lokomat^®^	45	20	3–5	4–6	Statistically significant difference (*p* < 0.001) and large effect size in GMFM in favor of the RAGT group.
Manikowska, F et al., 2021 [88]	CG CT EG RAGT + CT	EksoGT^®^	30–60	30	5	10 (2 wk work + 2 wk break)	Walking speed significantly improved (t2 vs. t3, *p* = 0.02) for group AS.
Peri, E. et al., 2017 [89]	CG_1_ TOP10 (CT) EG_1_ RAGT EG_2_ RAGT + TOP10EG_3_ RAGT + TOP4	Lokomat^®^	45	40	4 4 2 + 2 4 + 4	10 10 10 4	No differences among the 4 groups. Only RAGT and TOP groups obtained significant improvement in gross motor function.
Pool, D. et al., 2021 [90]	CG LT (TT) EG RAGT+LT	RT600^®^	60	18	3	6	No significant differences between the groups.
Romei, M. et al., 2012 [91]	CG TOP (CT) EG RAGT + TOP	Lokomat^®^	30	40	4 2 + 2	10	Both groups improved GMFM scores with no statistically significant differences. No improvement in their 6MinTW scores.
Sarham, RSM et al., 2014 [92]	CG CT EG RAGT	Lokomat Pro Version 4^®^	30–40	30	3	10	EG significantly improves stride length, cadence and gait speed (*p* < 0.001). CG does not show significant improvement.
Smania, N et al., 2011 [80]	CG CT EG RAGT + CT	Gait Trainer GT I^®^	40 30 + 10	10	5	2	Comparison between the groups shows statistically significant differences favoring the EG in gait speed (*p* < 0.001), 6MinWT (*p* = 0.015) and step length (*p* = 0.004).
Wallard, L. et al., 2017 [81]	CG CT EG RAGT	Lokomat^®^	40	20	5	4	Statistically significant differences favoring the EG in dimension D (*p* = 0.048) and dimension E (*p* = 0.026)
Wallard, L. et al., 2018 [82]	CG CT EG RAGT	Lokomat^®^	40	20	5	4	Significant differences were also found for the intergroup comparison in gait speed (*p* = 0.031), cadence (*p* = 0.043), step length (*p* = 0.042), step width (*p* = 0.022) and step width (*p* = 0.029).
Wu, M. et al., 2017 [83]	CG TT EG RAGT	3DCaLT^®^	30–40	18	3	6	RT significantly increases walking speed (*p* = 0.03) and a greater increase in 6MinWT over TT (*p* = 0.01).
Yazici, M et al., 201 [84]	CG CT EG RAGT + CT	Innowalk Pro^®^	30	36	3	12	No between-group analysis is performed but the within-group analysis of the EG shows significant changes in GMFM-88 (*p* < 0.001), GMFM-D (*p* = 0.003) and GMFM-E (*p* = 0.000) scores in the short term, and the first two are maintained in the long term.

Abbreviations: CG, Control Group; EG, Experimental Group; ss, Sessions; wk, Weeks; CT, Conventional Therapy; RAGT, Robotic Assisted Gait Training; TT, Treadmill Training; UC, Usual Care; PBWSTE, Partial Body Weight Supported Treadmill Exercise; ATE, Antigravity Treadmill Exercise; IE, individual exercise; TOP, Task-Oriented Physiotherapy.

**Table 4:** Results of methodological quality and risk of bias on the PEDro scale.

Study	1	2	3	4	5	6	7	8	9	10	11	Total
Ammann-Reiffer, C. et al., 2020 [78]	Yes	Yes	No	Yes	No	No	No	Yes	Yes	Yes	Yes	6
Aras, B. et al., 2019 [79]	Yes	Yes	Yes	Yes	No	No	No	Yes	No	Yes	Yes	6
Druzbicki, M. et al., 2013 [85]	y	Yes	No	Yes	No	No	Yes	No	No	Yes	Yes	5
Jin, LH. et al., 2020 [86]	Yes	Yes	No	Yes	No	No	Yes	Yes	No	Yes	Yes	6
Klobucká, S. et al., 2020 [87]	No	Yes	No	Yes	No	No	No	Yes	Yes	Yes	Yes	6
Manikowska, F. et al., 2021 [88]	Yes	Yes	No	Yes	No	No	No	Yes	No	Yes	Yes	5
Peri, E. et al., 2017 [89]	Yes	Yes	No	Yes	No	No	No	Yes	Yes	Yes	Yes	6
Pool, D. et al., 2021 [90]	Yes	Yes	Yes	Yes	No	No	Yes	Yes	Yes	Yes	Yes	8
Romei, M. et al., 2012 [91]	Yes	Yes	No	Yes	No	No	No	Yes	Yes	Yes	Yes	6
Sarham, RSM. et al., 2014 [92]	Yes	Yes	No	Yes	No	No	No	Yes	Yes	Yes	Yes	6
Smania, N. et al., 2011 [80]	Yes	Yes	Yes	Yes	No	No	Yes	Yes	No	Yes	Yes	7
Wallard, L. et al., 2017 [81]	Yes	Yes	No	Yes	No	No	Yes	No	No	Yes	Yes	5
Wallard, L. et al., 2018 [82]	Yes	Yes	No	Yes	No	No	No	No	No	Yes	No	3
Wu, M. et al., 2017 [83]	Yes	Yes	Yes	Yes	No	No	No	Yes	No	Yes	Yes	6
Yazici, M. et al., 201 [84]	Yes	No	No	Yes	No	No	No	Yes	Yes	Yes	Yes	5

Abbreviations: 1, Eligibility criteria; 2, Random allocation; 3, Concealed allocation; 4, Baseline comparability; 5, Blind subjects; 6, Blind therapists; 7, Blind assessors, 8. Adequate follow-up; 9, Intention-to-treat analysis; 10, Between-group comparisons; 11, Point estimates and variability. Note: Eligibility criteria item does not contribute to total score.

**Table 5:** Main findings in meta-analysis.

		Findings Summary										
		Effect Size						Heterogeneity		Publication Bias		
Variable (Post-Intervention Assessment)	Specific Comparison	K	N	N_s_	SMD	95% CI	*p*	Q (df)	*I*^2^ *(p)*	Egger *p*	Trim and Fill	
											Adj SMD	% var
Gait Speed	RAGT vs. TT	4	121	30.3	0.25	−0.15 to 0.64	0.22	11.5 (4)	51.3% (0.02)	0.3	0.19	24%
	RAGT vs. CT	3	58	19.3	0.56	0.03 to 1.1	0.04	2.52 (2)	20.6% (0.28)	0.65	0.56	0%
	RAGT + CT vs. CT	5	123	24.6	−0.1	−0.47 to 0.29	0.63	2.12 (3)	0% (0.55)	0.05	−0.18	100%
Step Length	RAGT vs. TT	3	81	27	0.1	−0.41 to 0.6	0.71	0.08 (2)	0% (0.96)	0.43	0.1	0%
	RAGT + CT vs. CT	3	79	26.3	0.2	−0.28 to 0.67	0.43	0.27 (2)	0% (0.87)	0.52	0.2	0%
Step width	RAGT + CT vs. CT	2	61	30.5	−0.28	−0.83 to 0.28	0.33	0.86 (1)	0% (0.35)	NP	NP	NP
Stride length	RAGT vs. TT	2	58	29	0.17	−0.46 to 0.8	0.6	0.27 (1)	0% (0.61)	NP	NP	NP
Walking distance	RAGT vs. TT	3	81	27	0.1	−1 to 1.2	0.86	0.96 (2)	0% (0.62)	0.14	0.1	0%
	RAGT vs. CT	2	60	30	2	0.36 to 3.65	0.017	3.79 (1)	32.3% (0.05)	NP	NP	NP
	RAGT + CT vs. CT	5	149	29.8	0.35	−0.51 to 1.2	0.43	0.05 (2)	0% (0.97)	0.12	0.42	20%
Cadence	RAGT vs. TT	2	58	29	0.09	−0.54 to 0.72	0.79	0.01 (1)	0% (0.92)	NP	NP	NP
	RAGT vs. CT	2	42	21	0.21	−0.4 to 0.82	0.5	0.01 (1)	0% (0.92)	NP	NP	NP
	RAGT + CT vs. CT	2	44	22	0.3	−0.31 to 0.92	0.33	0.3 (1)	0% (0.59)	NP	NP	NP
Standing ability	RAGT vs. TT	3	81	27	−0.01	−0.52 to 0.5	0.96	0.11 (2)	0% (0.95)	0.27	−0.01	0%
	RAGT vs. CT	3	90	30	−0.12	−0.61 to 0.36	0.62	4.22 (2)	52% (0.12)	0.01	0.32	100%
	RAGT + CT vs. CT	5	131	26.2	0.22	−0.13 to 0.56	0.21	0.19 (4)	0% (0.99)	0.88	0.22	0%
Walking, running and jumping abilty	RAGT vs. TT	3	81	27	0.11	−0.49 to 0.71	0.72	0.01 (2)	0% (0.99)	0.27	0.11	0%
	RAGT vs. CT	3	90	30	0.7	0.09 to 1.4	0.035	4.72 (2)	47% (0.05)	0.33	0.7	0%
	RAGT + CT vs. CT	5	131	26.2	0.11	−0.31 to 0.54	0.61	0.48 (4)	0% (0.97)	0.09	0.22	100%
Gross motor function	RAGT vs. TT	2	63	31.5	0.15	−0.36 to 0.65	0.57	0.05 (1)	0% (0.82)	NP	NP	NP
	RAGT + CT vs. CT	4	154	38.5	0.18	−0.2 to 0.56	0.36	0.42 (3)	0% (0.93)	0.2	0.23	22%
Funct. indep.	RAGT + CT vs. CT	2	42	21	0.14	−0.46 to 0.75	0.64	0.08 (1)	0% (0.77)	NP	NP	NP

Abbreviations: K, Number of comparisons; N, Total sample size; Ns, Participants per study; SMD, Standardized Mean Difference; 95% CI, 95% Confidence Interval; *p*, *p*-value; Q, Q-test; df, degree of freedom; *I*^2^, Degree of Inconsistency; Adj, Adjusted; % var, Percentage of variation; RAGT, Robotic-assisted gait training; TT, Treadmill Training; CT, Conventional Therapy; Funct. Indep, Functional Independence; NP, Not possible to calculate.

## Data Availability

Not applicable.
